# Endogenous endophthalmitis secondary to bacterial meningitis from Neisseria Meningitidis: a case report and review of the literature

**DOI:** 10.1186/1757-1626-2-149

**Published:** 2009-10-07

**Authors:** Konstantinos Balaskas, Dora Potamitou

**Affiliations:** 1 Ophthalmology Department, "Archbishop Makarios III" Hospital, Nicosia, Cyprus

## Abstract

**Background:**

To report the case of a patient with endogenous endophthalmitis secondary to meningitis from Neisseria Meningitidis with early detection and good visual recovery.

**Case report:**

A 20-year old patient treated for meningitis was referred to us for vision blurring of his left eye. Unilateral endogenous panophthalmitis was diagnosed with visual acuity hand movement at 1 meter and vitreous sample was obtained for culture. The patient was already receiving intravenous ceftriaxone and dexamethasone. Ceftazidime was injected intravitreally. Four months later visual acuity improved to 4/10 on the Snellen's scale but the development of extensive fibrous strands and the risk for vessel rupture led to vitreous surgery. One year later the visual acuity is stable at 5/10.

**Conclusion:**

Endogenous endophthalmitis constitutes a rare complication of bacterial meningitis and its prompt diagnosis and administration of intravitreal antibiotics could lead to a more favorable visual prognosis.

## Case Report

A 20-year old patient of Caucasian origin, born in Greece, was admitted to the medical ward with signs and symptoms of bacterial meningitis. The patient reported fever, headache and vomiting for the previous two days. Treatment administered consisted of intravenous ceftriaxone 2 gr daily and dexamethasone 4 mg every six hours. Cerebrospinal fluid was obtained for biochemistry and culture with findings: leukocytes 1320/dl (68% neutrophils), glucose 1 mg/dl, protein 223, 1 mg/dl and the immediate gram stain revealed gram negative diplococci. Blood culture (Bactec 9240 instrument; Becton, Dickinson and Company, Sparks, MD) confirmed the diagnosis of meningitis from Neisseria Meningitidis type B four days later.

**Figure 1:**
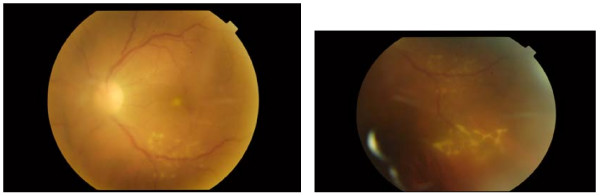
**Caption on the day of diagnosis**. Vitreus inflammation obscuring clear view of fundus. Foveal infiltrate. Periphlebitis.

**Figure 2:**
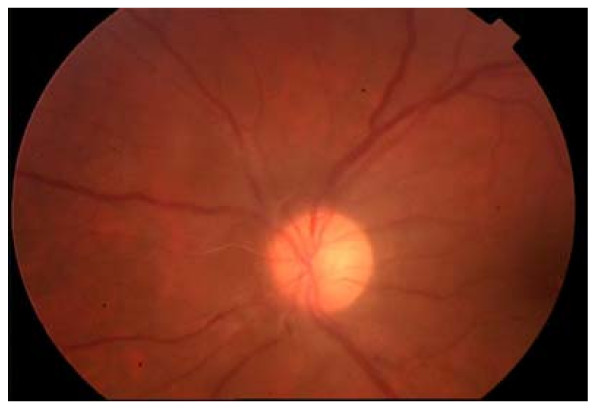
**Caption one month later**. Vitreus has cleared. Fibrous tissue beginning to develop around the disc. VA at 3/10

**Figure 3:**
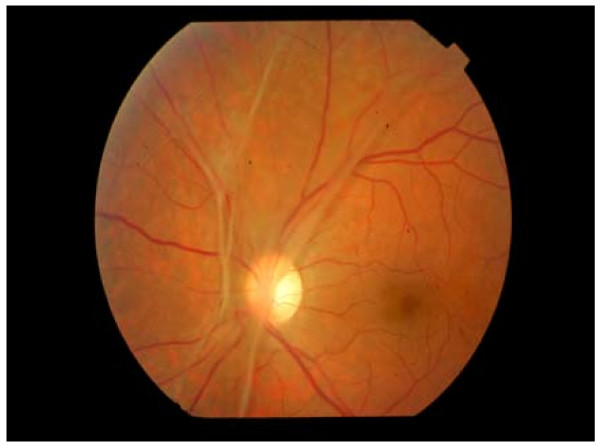
**5 months later extensive fibrous strands have been formed**. Vitrectomy is considered to prevent the likelihood of blood vessel rupture and intravitreal haemorrhage. VA stabilised at 4/10.

On the second day of treatment the patient complained for severe blurring of vision of his left eye and was referred for ophthalmologic evaluation. Vision acuity was hand movement at 1 meter; there was severe inflammatory reaction in the anterior chamber (aqueous flair and leukocytes) and vitreous opacity grade II in the left eye. Intraocular pressure was 15 mmHg. Fundus indirect ophthalmoscopy revealed sheathing of the inferior temporal branch of the central retinal vein, infiltrates at the inferior temporal quadrant of the mid periphery of the retina and an infiltrate in the subfoveal area [figure 1]. The diagnosis of endogenous panophthalmitis was made and a vitreal tap was performed in order to isolate the responsible pathogen. The initial vitreous aspirates were sent for gram stain, culture (bacterial and fungus) and antibacterial sensitivity. The samples were subjected to Gram stain, Periodic Acid Schiff stain and calcofluor stains for direct microscopy under light and fluorescent microscope. Cultures were obtained on 5% blood and chocolate agar (any organism), anaerobic thioglycollate broth and Sabourauds agar (fungi). Gram stain revealed gram negative intracellular organisms. Culture came back negative. An intravitreal injection of ceftazidime (2 mg/0.1 ml) was performed as well as daily subconjactival injections with ceftazidime. On the seventh day intravenous treatment was ceased and oral administration of prednisolone 16 mg every 8 hours was initiated and was continued for two months tapering progressively. Intraocular inflammation gradually improved. One month later vitreous had cleared significantly and visual acuity was 3/10 on the Snellen's scale [Figure 2]. On the fourth month visual acuity was stable at 4/10, inflammation had receded but the presence of preretinal fibrous strands which caused elevation of an inferior vessel branch prompted us to perform vitrectomy in order to avert the possibility of intravitreous hemorrhage [Figure 3]. One year later visual acuity has stabilized at 5/10 and the patient feels quite content with the outcome.

## Discussion

Endogenous endophthalmitis constitutes a potentially devastating intraocular inflammation, caused by the migration of the pathogen from a distant primary site of infection to the eye where it crosses the blood-ocular barrier. It can occur at any age, ranging from 1 week to 85 years. Bilateral involvement is seen in 14 to 25% of patients and reports have shown a higher incidence of involvement of right eye versus left eye [1] in patients with unilateral disease. Before the widespread use of antibiotics the incidence of endophthalmitis secondary to bacterial meningitis was significant [2], though it has become very rare today.

The majority of patients with endogenous endophthalmitis suffer from an underlying disease. Predisposing factors are immunocompromise, intravenous drug abuse and prolonged stay in intensive care [3]. In several studies diabetes mellitus was shown to be the most common association (80-90% in one series). Fungi and bacteria are blamed for this condition with the same frequency. In different published series, fungal organisms are responsible for more than half of the cases of endogenous endophthalmitis with candida albicans being the commonest pathogen (75-80%) [4]. In patients with candidemia the occurrence of endogenous endophththalmitis ranges from 0-45% in the literature [5]. As regards to bacterial endogenous endophthalmitis, gram negative organisms are responsible for the majority of cases in East Asia, but gram positive organisms are more often encountered in North America and Europe. The most common gram positive organisms are group B streptococci, staphylococcus aureus, streptococcus pneumoniae and listeria monocytogenes. The most common gram negative organisms include Klebsiella spp., Escherichia coli, Pseudomonas aeruginosa and Neisseria meningitidis. [6] In a literature review it was concluded that within the east Asian population the patient at greatest risk for endogenous endophthalmitis is a diabetic patient with Klebsiella spp hepatobilary infection, whereas among the Caucasian population it is most common among predisposed patients with gram positive bacteremia in the context of endocarditis or skin/joints infections [7].

The identification of the responsible pathogen is essential for effective treatment. Debate exists over the efficacy of aspiration of intraocular material for endogenous endophthalmitis. Positive culture rates vary in the published literature (24-95%). In one study, vitreous cultures gave the highest positive results (74%), followed by blood cultures (72%) [8]. It is important that several culture media are available and if candida is suspected the sample must be centrifuged before cultured [9]. The use of a universal bacterial PCR can help detect the causative organism, especially, as in our case, for Neisseria meningitidis which is hard to isolate in a culture of an intraocular sample [10].

The outcome of endogenous endophthalmitis is generally worse than exogenous endophthalmitis because of the more aggressive pathogens typically involved with this condition (i.e., more virulent organisms) and because of compromised host immunity and delay in diagnosis. The prognosis appears to also be related to the patient's underlying health conditions, with worsened outcomes among diabetic patients. The prognosis varies considerably with respect to the responsible micro organism. The visual acuity at the time of diagnosis, the causative agent and the degree of vitreous opacity are the main prognostic factors for the outcome.

Prompt administration of intravenous antibiotic therapy is the cornerstone in the acute management of endogenous endophthalmitis. Role of intravitreal antibiotic injections and vitrectomy is under debate, but recent reports suggested that cases of marked intraocular infection (vitritis preventing visibility of optic nerve head or macula) should be managed similarly to cases of acute postoperative exogenous endophthalmitis. In a study, 12 eyes with endogenous endophthalmitis were given intravitreal antibiotics and subsequently all eyes with fungal endophthalmitis (3 eyes) underwent vitrectomy with injection of amphotericin B. In this study it was found that there was a definite improvement in degree of inflammation and visual acuity after vitrectomy and intravitreal injection of antibiotics [11].

In the case presented here the patient was a young man without any predisposing factors. The pathogen responsible for the infection was not isolated in the vitreal sample; however the gram stain, the severity of the intraocular inflammation and the positive blood cultures for neisseria meningitidis are in favor of the diagnosis of endogenous endophthalmitis secondary to the bacterial meningitis. Ocular implication in the context of meningitis from Neisseria meningitidis is exceptional. In a series of 28 cases there was only one incident attributable to neisseria meningitidis [12]. In the literature there are few reports of atypical endogenous endophthalmitis from neisseria meningitidis presenting as anterior uveitis [13]. In two other cases the pathogen was detected by PCR in the intraocular fluid, without the presence of associated meningitis [14].

Bearing in mind the poor prognosis of the condition with only 30% of all eyes obtaining counting fingers or better final visual acuity and 16% being enucleated, the final outcome in our case is encouraging.

## Conclusion

The rarity of endogenous endophthalmitis secondary to bacterial meningitis should not diminish our degree of awareness for this condition in immunocompetent patients. Early diagnosis and prompt administration of intravenous and intravitreal antibiotics can greatly improve the prognosis and help the patient retain a considerable degree of useful vision. In our case the early aggressive treatment, topical and systematic, led to an unexpected improvement of visual acuity in a condition the sombre prognosis of which has remained virtually unchanged the last five decades.

## Competing interests

The authors declare that they have no competing interests.

## Authors' contributions

KB: Main Author, DP: Supervising Consultant. All authors read and approved the final manuscript.

## Consent

Written informed consent was obtained from the patient for publication of this case report and accompanying images. A copy of the written consent is available for review by the journal's Editor-in-Chief.

## References

[B1] Das T Kunimoto DY Sharma S Jalali S Majji AB Nagaraja Rao T Relationship between clinical presentation and visual outcome in postoperative and posttraumatic endophthalmitis in south central India Indian J Ophthalmol 2005 53 5 16 10.4103/0301-4738.15298 15829741

[B2] Lazar N Early ocular complications of epidemic meningitis Arch Ophthalmol 1936 16 847 56

[B3] Ness T Endogenous endophthalmitis Ophthalmologe 2007 104 11 935 9 10.1007/s00347-007-1650-6 17943291

[B4] Schiedler V Scott IU Flynn HW Jr Davis JL Benz MS Miller D Culture-proven endogenous endophthalmitis: Clinical features and visual acuity outcomes Am J Ophthalmol 2004 137 725 31 15059712 10.1016/j.ajo.2003.11.013

[B5] Khan FA Slain D Khakoo RA Candida endophthalmitis: focus on current and future antifungal treatment options Pharmacotherapy 2007 27 12 1711 21 10.1592/phco.27.12.1711 18041891

[B6] Jackson TL Eykyn SJ Graham EM Stanford MR Endogenous bacterial endophthalmitis: a 17-year prospective series and review of 267 reported cases Surv Ophthalmol 2003 48 4 403 23 10.1016/S0039-6257(03)00054-7 12850229

[B7] Wong JS Chan TK Lee HM Chee SP Endogenous bacterial endophthalmitis: an east Asian experience and a reappraisal of a severe ocular affliction Ophthalmology 2000 107 8 1483 91 10.1016/S0161-6420(00)00216-5 10919895

[B8] Donahue SP Kowalski RP Jewart BH Friberg TR Vitreous cultures in suspected endophthalmitis. Biopsy or vitrectomy? Ophthalmology 1993 100 452 5 8479699 10.1016/s0161-6420(93)31623-4

[B9] Ness T Serr A Diagnostics for endophthalmitis Klin Monatsbl Augenheilkd 2008 225 1 44 9 10.1055/s-2008-1027132 18236369

[B10] Quintyn JC Poupelin S Fajoles-Vasseneix C Brasseur G Meningococcus endophthalmitis without meningitis J Fr Ophtalmol 2006 29 9 e24 French 17114987

[B11] Tulsi Keswani 1 Vijay Ahuja 2 Manish Changulani 3 Evaluation of outcome of various treatment methods for endogenous endophthalmitis Indian journal of medical sciences 2006 60 11 454 460 10.4103/0019-5359.27972 17090866

[B12] Okada AA Johnson RP Liles WC D'Amico DJ Baker AS Endogenous bacterial endophthalmitis. Report of a ten-year retrospective study Ophthalmol 1994 101 832 8 8190467

[B13] Chhabra MS Noble AG Kumar AV Mets MB Neisseria meningitidis endogenous endophthalmitis presenting as anterior uveitis J Pediatr Ophthalmol Strabismus 2007 44 5 309 10 17913177 10.3928/01913913-20070901-09

[B14] Kerkhoff FT Zee A van der Bergmans AM Rothova A Polymerase chain reaction detection of Neisseria meningitidis in the intraocular fluid of a patient with endogenous endophthalmitis but without associated meningitis Ophthalmology 2003 110 11 2134 6 10.1016/S0161-6420(03)00834-0 14597520

